# Classification of Chondrosarcoma: From Characteristic to Challenging Imaging Findings

**DOI:** 10.3390/cancers15061703

**Published:** 2023-03-10

**Authors:** Jun-Ho Kim, Seul Ki Lee

**Affiliations:** 1 Department of Orthopaedic Surgery, Center for Joint Diseases, Kyung Hee University Hospital at Gangdong, Seoul 05278, Republic of Korea; 2 Department of Radiology, St. Vincent’s Hospital, College of Medicine, The Catholic University of Korea, Seoul 06591, Republic of Korea

**Keywords:** chondrosarcoma, classification, 2020 World Health Organization classification of tumors of soft tissue and bone, atypical cartilaginous tumor, high-grade chondrosarcoma, plain radiograph, computed tomography, magnetic resonance imaging

## Abstract

**Simple Summary:**

Chondrosarcomas are a very heterogeneous group of cartilage-forming tumors that comprise approximately one-third of all malignant bone tumors. The World Health Organization classifies chondrosarcomas as benign, intermediate, or malignant cartilaginous tumors. Clinical management is guided by characteristic imaging findings and histopathological grade. However, the differentiation between enchondromas and low-grade chondrosarcomas and between low-grade and high-grade chondrosarcomas is challenging for radiologists and pathologists. Many potentially helpful advanced imaging modalities exist for diagnosing chondroid tumors and multidisciplinary discussions of all modalities should be combined when making treatment decisions.

**Abstract:**

Chondrosarcomas can be classified into various forms according to the presence or absence of a precursor lesion, location, and histological subtype. The new 2020 World Health Organization (WHO) Classification of Tumors of Soft Tissue and Bone classifies chondrogenic bone tumors as benign, intermediate (locally aggressive), or malignant, and separates atypical cartilaginous tumors (ACTs) and chondrosarcoma grade 1 (CS1) as intermediate and malignant tumors. respectively. Furthermore, the classification categorizes chondrosarcomas (including ACT) into eight subtypes: central conventional (grade 1 vs. 2–3), secondary peripheral (grade 1 vs. 2–3), periosteal, dedifferentiated, mesenchymal, and clear cell chondrosarcoma. Most chondrosarcomas are the low-grade, primary central conventional type. The rarer subtypes include clear cell, mesenchymal, and dedifferentiated chondrosarcomas. Comprehensive analysis of the characteristic imaging findings can help differentiate various forms of chondrosarcomas. However, distinguishing low-grade chondrosarcomas from enchondromas or high-grade chondrosarcomas is radiologically and histopathologically challenging, even for experienced radiologists and pathologists.

## 1. Introduction

Chondrosarcomas are malignant tumors that produce a chondroid (cartilaginous) matrix [1,2]. They can be classified as either primary or secondary. Primary chondrosarcomas, which arise de novo, are the third most common primary malignant tumors of the bone after myelomas and osteosarcomas and account for 20–27% of all primary malignant bone tumors [1]. Conversely, secondary chondrosarcomas are associated with pre-existing cartilaginous lesions, such as enchondroma or osteochondroma [3,4]. Chondrosarcomas can also be classified based on the osseous location in which they arise; namely, central (within the intramedullary cavity), peripheral (within the cartilage cap of a pre-existing osteochondroma), or periosteal (juxtacortical; on the surface of the bone) [5]. Further classification of chondrosarcomas is based on histological subtypes, including conventional (grades 1–3), clear cell, mesenchymal, and dedifferentiated [6]. Finally, the 2020 World Health Organization (WHO) classification categorizes chondrosarcomas into eight subtypes: central conventional (grade 1 vs. 2–3), secondary peripheral (grade 1 vs. 2–3), periosteal, dedifferentiated, mesenchymal, and clear cell [7]. The characteristic imaging features of numerous categories of chondrosarcomas may aid in accurate diagnosis and classification. Radiography can support the diagnosis of chondroid tumors as enchondromas with characteristic findings including typical chondroid matrix mineralization [8]. Computed tomography (CT) and magnetic resonance imaging (MRI) can reveal imaging features of malignancy to distinguish between chondrosarcomas and enchondromas [8]. This review article summarizes the various classifications of chondrosarcomas and provides the characteristic to challenging imaging findings to differentiate among the various forms of chondrosarcoma.

## 2. 2020 WHO Classification of Chondrosarcomas

**Figure 1:**
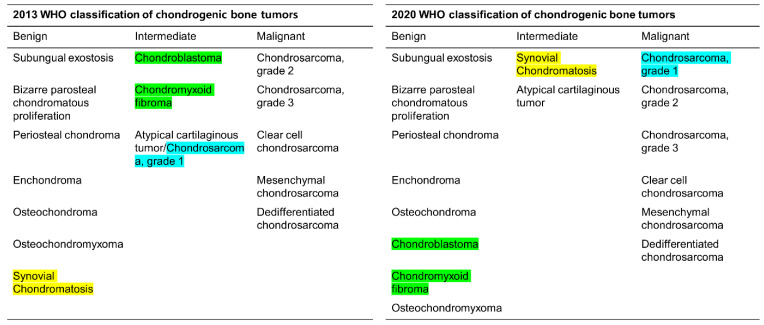
Comparison of the 2013 and 2020 World Health Organization (WHO) classifications of chondrogenic bone tumor. Diseases highlighted are those that are subject to change from 2013 to 2020 WHO classification.

The 2020 WHO classification categorizes chondrogenic bone tumors as benign, intermediate (locally aggressive), or malignant (Figure 1) [7]. The term “atypical cartilaginous tumor (ACT)”, which was first introduced in the 2013 WHO classification, refers to low-grade chondrosarcomas located in the appendicular skeleton (long and short tubular bones) that are considered the intermediate group (chondrosarcoma grade 0.5) [9]. Other chondrosarcomas are assigned to the malignant group. It is important to note that “chondrosarcoma grade 1 (CS1)” is histologically the same as ACT but is assigned to the malignant group; CS1 should be applied separately to tumors of the axial skeleton (including the pelvic bones and the skull base). Chondrosarcomas located in the axial skeleton have a worse outcome and require more aggressive treatment compared to those in the appendicular skeleton [7,10,11,12].

Finally, the 2020 WHO classification categorizes chondrosarcomas (including ACT) into eight subtypes (Table 1): central conventional (grade 1 vs. 2–3), secondary peripheral (grade 1 vs. 2–3), periosteal, dedifferentiated, mesenchymal, and clear cell chondrosarcoma [7]. We discuss four stages used to determine the classification of chondrosarcomas, as well as the characteristic to challenging features of various chondrosarcomas.

### 2.1. First Stage: Histological Grading 

The biological behavior of chondrosarcomas is graded as 1 to 3 based on nuclear size, staining pattern (hyperchromasia), mitotic activity, and cellularity degree [14]. CS1 refers to low-grade tumors containing chondrocytes with small dense nuclei, although some slightly enlarged nuclei (>8 μm) and a few multinucleated cells (most commonly binucleated) may be present [1]. The stroma is predominantly chondroid with sparse or absent myxoid areas [1]. Chondrosarcoma grade 2 (CS2) tumors are intermediate-grade tumors containing less chondroid matrix and an increased cellular portion compared to CS1 tumors [1]. Chondrocyte nuclei are enlarged, either vesicular or hyperchromatic, and are also binucleated and multinucleated [1]. The stroma is frequently myxoid [1]. Chondrosarcoma grade 3 (CS3) tumors are high-grade tumors exhibiting greater cellularity than CS1 and CS2 tumors and nuclear pleomorphism with sparse or absent chondroid matrix [1]. The nuclei are typically vesicular, often spindle-shaped, and may be 5–10-fold larger than normal [1]. The non-mineralized tissue in chondrosarcomas has high water content, varying histologically from mature hyaline cartilage to a more myxoid stroma [1]. The edges of chondrosarcomas are characterized by chondroid tissue invading the trabecular bone [15]. Once this morphological feature has been identified, the degree of cellularity is used to determine the chondrosarcoma grade [1]. Invasion of the endosteal surface marks the beginning of extraosseous extension as the first step toward high-grade chondrosarcoma [10]. 

Most chondrosarcomas are conventional, with 60% classified as CS1 or CS2 [16]. Conventional chondrosarcomas tend to occur in older people, and more than 50% of patients are >50 years of age [13]. These chondrosarcomas are referred to as central chondrosarcomas. The 5-year survival rate is 88% for patients with CS1 and 57% for patients with CS2 and CS3 with local recurrence and metastasis rates of 20% and 14%, respectively [17]. The most common skeletal location for conventional chondrosarcomas is the long tubular bone, accounting for approximately 45% of cases [1,18,19]. The femur is the single most commonly affected long bone, representing approximately 20–35% of cases, while the upper extremity is involved in 10–20% of cases, most frequently the proximal humerus [1,18,19]. Long tubular bone lesions most commonly involve the metaphysis (49% of cases) [15]. Conventional chondrosarcomas can also occur in flat bones such as the pelvic bones; however, higher-grade tumors more frequently occur in the axial skeleton than in the appendicular skeleton. For instance, the prevalence of CS2-3 in the iliac bone is 70% vs. 45% in the femur [20].

**Figure 2:**
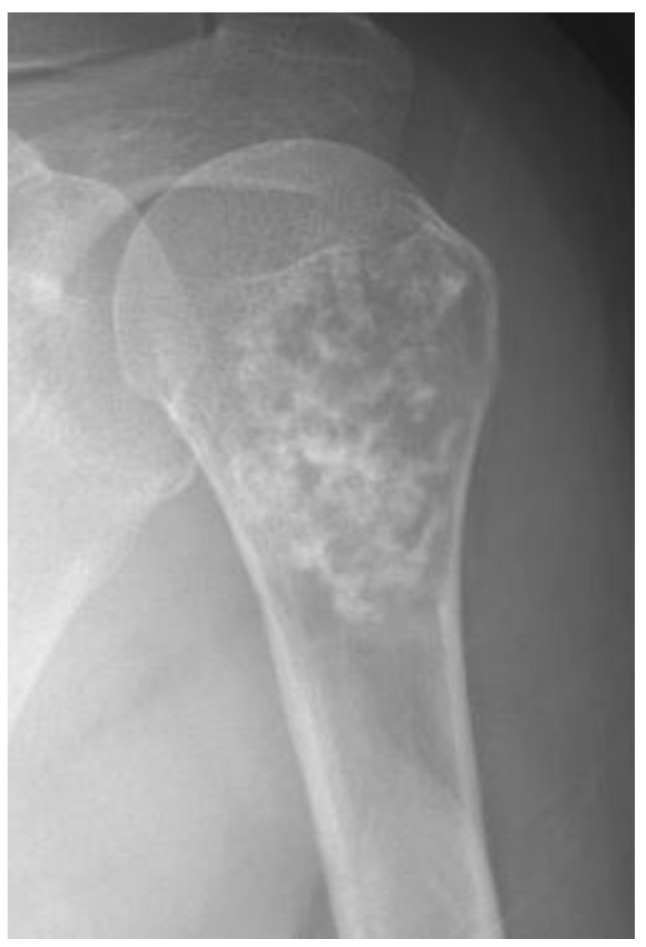
**Atypical cartilaginous tumor of the humerus in a 59-year-old woman.** Anteroposterior shoulder radiograph shows a mixed lytic and sclerotic lesion in the humerus. The sclerotic component represents typical chondroid ring-and-arc calcification.

Radiographs of conventional chondrosarcomas typically reveal a mixed lytic and sclerotic appearance [1,5]. The sclerotic areas represent chondroid matrix mineralization, which is seen in 60–78% of lesions [1]. Well-differentiated tumors tend to have a characteristic ring-and-arc pattern (Figure 2), whereas higher-grade chondrosarcomas often contain relatively less matrix mineralization and have a more amorphous or stippled appearance [1,5,19,21,22,23]. It is vital to differentiate benign from malignant cartilage tumors; increased biological activity presents as deep and extensive endosteal scalloping as an attempt of tumor cell extension to a second compartment [1].

**Figure 3:**
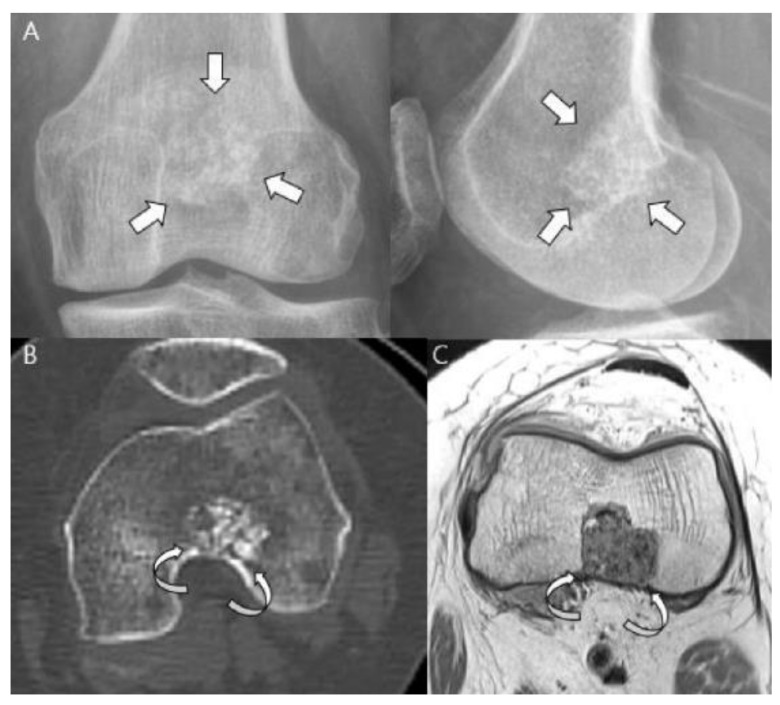
**Atypical cartilaginous tumor of the distal femur in a 50-year-old woman.** (**A**) Anteroposterior and lateral radiographs reveal a mixed lytic and sclerotic lesion in the distal femur (arrows) with typical ring-and-arc calcifications. (**B**) Computed tomography and (**C**) axial T2-weighted image demonstrate a lobulated chondroid tumor with deep endosteal scalloping (curved arrows) despite the small tumor size (1.7 cm).

**Figure 4:**
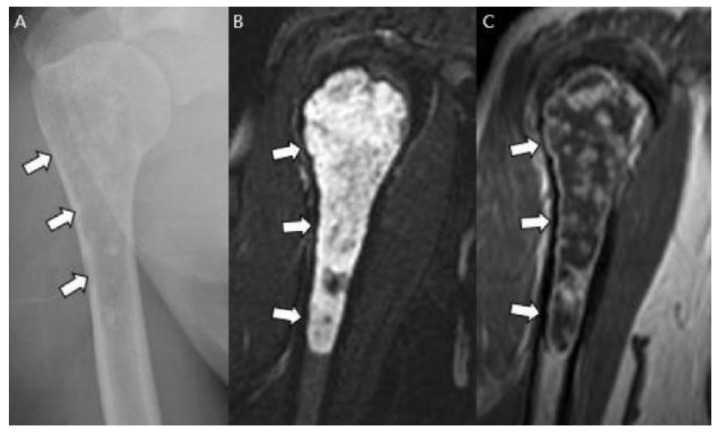
**A typical cartilaginous tumor of the humerus in a 43-year-old woman.** (**A**) Radiographs reveal a mixed lytic and sclerotic lesion in the humerus (arrows) with typical ring-and-arc calcifications. (**B**) Coronal T2-weighted image with fat suppression and (**C**) T1-weighted enhanced image demonstrate a lobulated chondroid tumor with longitudinal endosteal scalloping (arrows) along the 9 cm length of the tumor.

Sensitive radiographic features differentiating enchondromas from chondrosarcomas include deep endosteal scalloping ≥2/3 of the normal cortical thickness [5,15] (Figure 3). Extensive longitudinal endosteal scalloping over ≥2/3 of the lesion length is also strong evidence of chondrosarcoma (although a somewhat less reliable criterion) [1,24] (Figure 4).

**Figure 5:**
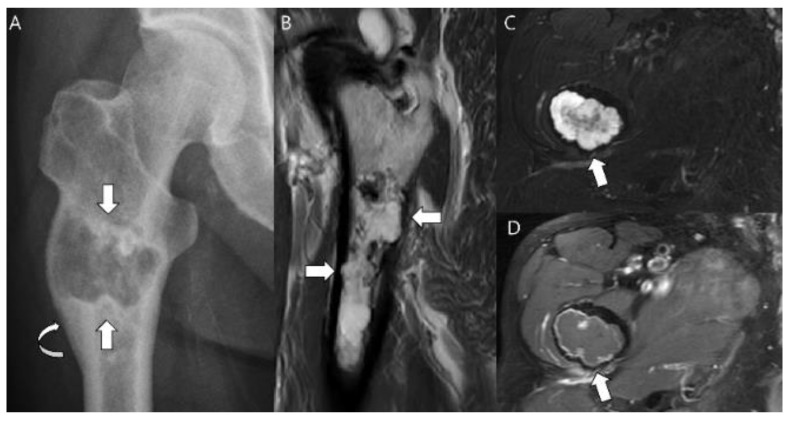
**Chondrosarcoma grade 2 of the proximal femur in a 71-year-old man.** (**A**) Anteroposterior radiograph reveals a lytic lesion in the proximal femur (arrows) resulting in cortical thickening and periosteal reaction (curved arrow). (**B**) Sagittal T2-weighted image shows a markedly high-signal lesion with deep endosteal scalloping (arrows). (**C**) Axial T2-weighted image with fat suppression and (**D**) axial T1-weighted enhanced image demonstrates a lobulated chondroid tumor with focal bone expansion (arrows).

Chondrosarcomas frequently grow slowly, and the cortex responds to maintain the tumor in the medullary cavity. This attempt leads to the maintenance of a chondrosarcoma margin presenting as cortical remodeling, cortical thickening, and periosteal reaction [1,5] (Figure 5). 

**Figure 6:**
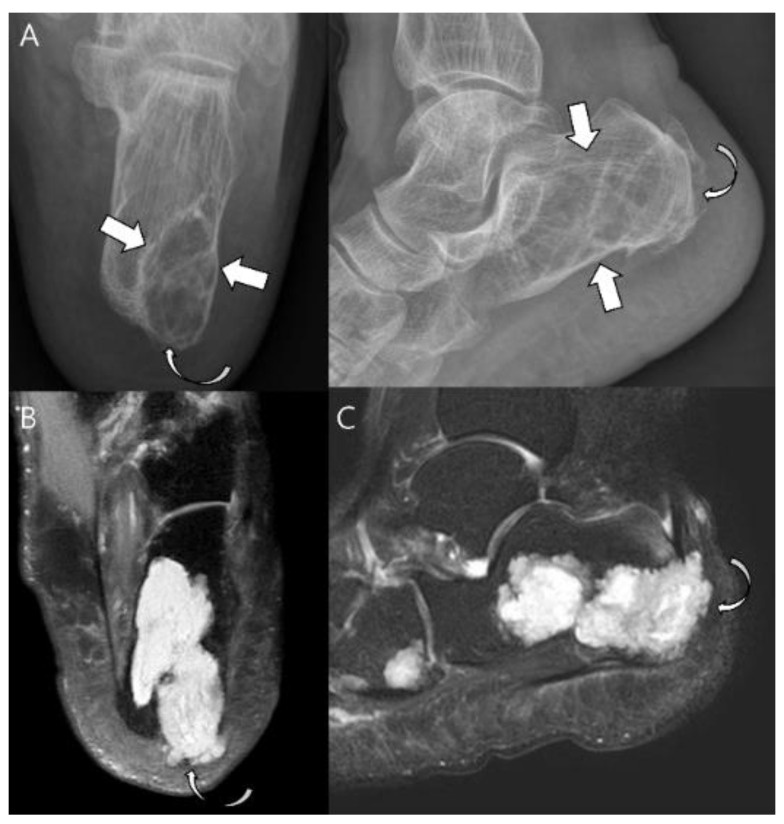
**Atypical cartilaginous tumor of the calcaneus in a 74-year-old woman.** (**A**) Plain radiographs reveal a lytic lesion in the calcaneus (arrows) with a partially destructed cortex (curved arrow). (**B**) Axial and (**C**) sagittal T2-weighted images with fat suppression show a lesion with marked high-signal intensity with focal extraosseous soft tissue extension (curved arrows).

**Figure 7:**
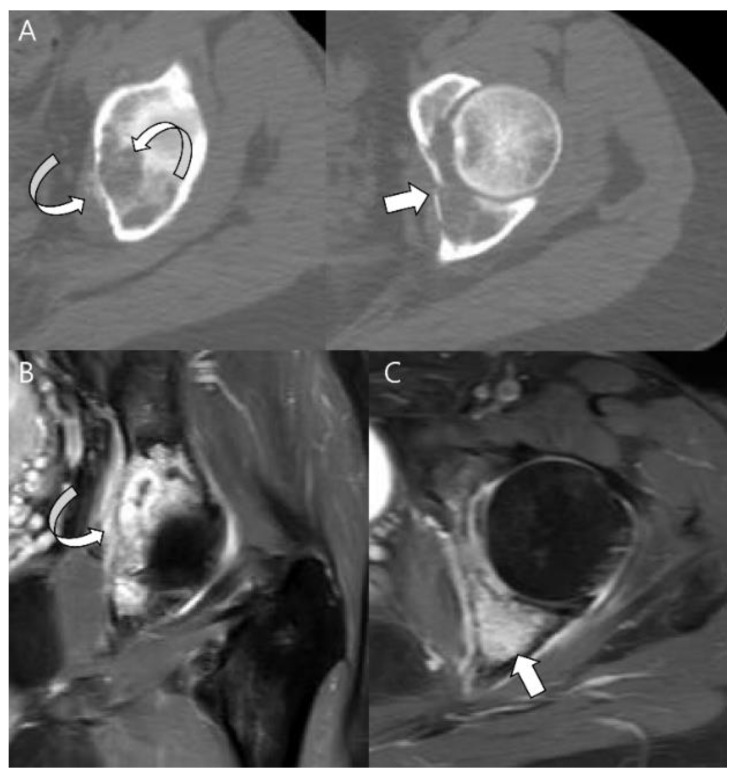
**High-grade conventional chondrosarcoma of the acetabulum in a 49-year-old woman.** (**A**) Axial CT scans reveal cortical breakage (thin arrow) with extraosseous extension containing matrix mineralization (curved arrow) in the left acetabulum. (**B**) Axial and (**C**) axial T1-weighted enhanced images with fat suppression show diffusely enhancing intraosseous (arrow) and extraosseous tumor components (curved arrow).

Cortical destruction and soft tissue masses are further findings that can indicate an aggressive process with a perfect specificity of 100% [8] (Figure 6). A more aggressive moth-eaten and permeative bone appearance with more ill-defined margins may be seen in higher-grade chondrosarcomas and is frequently associated with mesenchymal and dedifferentiated subtypes [1,18]. CT allows the optimal detection of matrix mineralization, particularly when it is subtle or in a complex anatomic area, in addition to the accurate evaluation of the length and depth of endosteal scalloping [1,15,24]. Cortical response or cortical destruction with extraosseous soft tissue extension can also be well visualized by CT [15,24,25]. The enhanced CT findings for chondrosarcoma include a mild peripheral rim and septal enhancement [1,24]. MRI is the best method for evaluating the extent of marrow replacement and soft tissue extension [1,24]. Conventional chondrosarcomas have water-rich hyaline cartilage, which presents as a bright signal surrounded by low-signal septa on T2-weighted images (T2WI) [15]. Areas of matrix mineralization have a low signal in all MR pulse sequences [15]. This feature often creates marked heterogeneity in T2WI [1]. On T1-weighted images (T1WI), marrow-replacing lesions show a low-to-intermediate signal with possible entrapped areas of pre-existing fat marrow, presenting with high signal intensity on T1WI [1]. Soft tissue extension is well demonstrated on MRI and the characteristics of soft tissue extension are identical to those of the intraosseous component [1,24]. The contrast enhancement pattern is typically mild in degree and peripheral and septal in pattern [1]. Higher-grade lesions appear, with larger soft tissue masses showing more prominent diffuse or nodular contrast enhancement [1]. Higher-grade conventional chondrosarcomas occur more frequently in the axial skeleton. The prevalence of CS2 and CS3 in the iliac bone is 70%, with a predilection for the area around the previous region of the triradiate cartilage (Figure 7). For comparison, the prevalence of CS2 and CS3 in the entire femur is 45% [20].

### 2.2. Second Stage: Primary vs. Secondary

Chondrosarcomas arising de novo are called primary chondrosarcomas (>90%), of which ≥80% are conventional (see Section 2.1) [16]. Conversely, chondrosarcomas superimposed on pre-existing benign cartilaginous neoplasms such as enchondromas or osteochondromas, those complicating enchondromatosis (Ollier’s disease, Maffucci syndrome), and hereditary multiple exostoses (HME) are referred to as secondary chondrosarcomas (<10%) [1,5,17]. Their reported incidence rates are 0.4% to 2.2% in patients with solitary osteochondroma or enchondroma [17] and increase to 27.3% in patients with HME [3,26,27], 30–50% in patients with Ollier’s disease, and up to 100% in patients with Maffucci syndrome [28,29,30]. Enchondromas are considered precursor lesions for ‘secondary central chondrosarcomas’, while osteochondromas are considered precursor lesions for ‘peripheral chondrosarcomas’. The terms ‘central’ and ‘peripheral’ relate to the location of the tumor in the affected bone [13,17]. Underlying genetic differences exist between primary and secondary chondrosarcomas and induce clinical variations in presentation and behavior [31]. Patients with secondary chondrosarcomas are generally younger than those with primary chondrosarcomas, with a mean age of 34 years. The tumors are also generally low-grade [17,31,32]. Changes in clinical symptoms in patients with known precursor lesions herald the development of chondrosarcomas [5,17]. The most common site of involvement is the pelvis, followed by the proximal femur. The scapula and proximal humerus are also relatively common sites [31].

**Figure 8:**
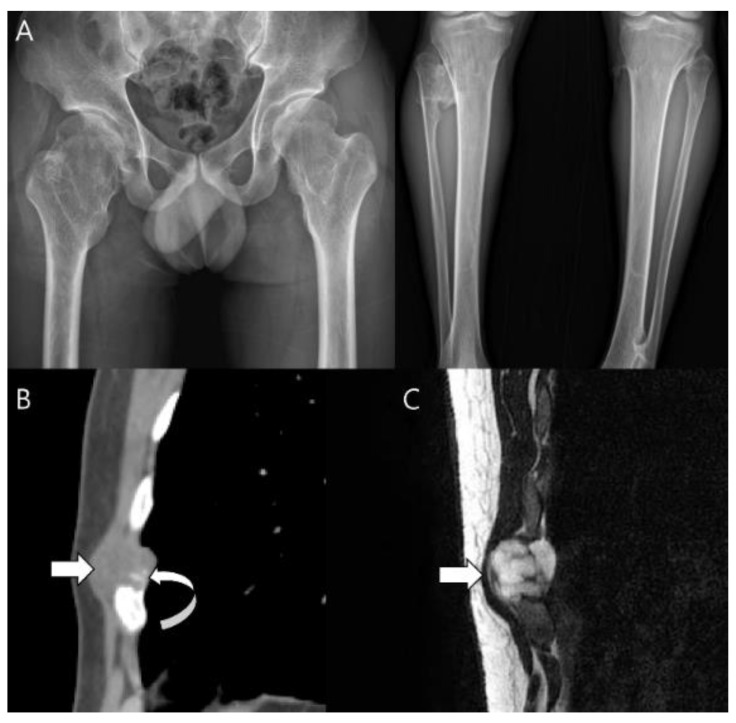
**Secondary peripheral chondrosarcoma of the rib in a 34-year-old man.** (**A**) Hip and tibial plain radiographs reveal underlying multiple exostoses. (**B**) Sagittal CT scan shows a lobulated mass with soft-tissue density (arrow) arising from the rib containing matrix mineralization (curved arrow), suggestive of a cartilage cap of sessile osteochondroma. (**C**) Sagittal T2-weighted image shows a mass of 2.3 cm in thickness with high signal intensity (arrow).

Secondary peripheral chondrosarcomas occur in the cartilage cap, and the diagnosis of malignant transformation depends on the measurement of cartilage cap thickness [33,34,35]. The radiographic features of malignant transformation include (1) growth of a previously unchanged osteochondroma in a skeletally mature patent; (2) irregular or indistinct lesion surface; (3) focal areas of osteolysis within the osseous component of the lesion; (4) erosion or destruction of the adjacent bone; and (5) a significant soft tissue mass containing scattered or irregular calcifications [36]. The thickness of the cartilage cap can be assessed critically by CT and MRI [33,35]. Bernard et al. recently concluded that a cartilage cap thickness > 2 cm strongly suggested malignant transformation of osteochondroma in skeletally mature patients [37] (Figure 8). The MRI appearance of chondrosarcoma arising from the cartilage cap is as expected for well-differentiated hyaline chondral tissue, with low signal on T1WI and markedly high signal on T2WI, showing peripheral and septal enhancement with a lobular growth pattern. Matrix mineralization appears as punctate or curvilinear low-signal foci [34,35]. Some authors have stressed the qualitative evaluation of the cartilage cap rather than the absolute measurement of cartilage cap thickness. Irregularity of the surface of the cartilage cap may reflect an increase in the invasive nature of the tumor [31].

**Figure 9:**
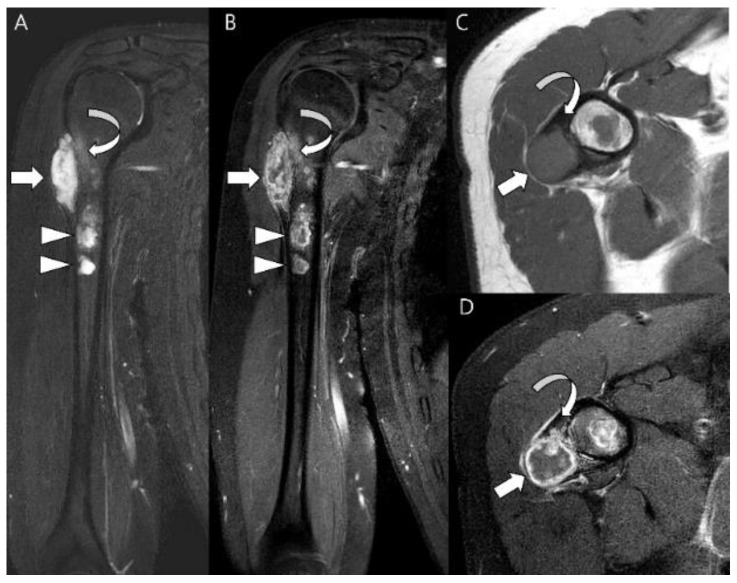
**Secondary central chondrosarcoma of the humerus in a 19-year-old man.** (**A**) Coronal T2-weighted image and (**B**) coronal T1-weighted enhanced images with fat suppression show multiple intramedullary chondroid tumors (arrowheads) with peripheral and septal enhancement in the humerus, suggesting enchondromatosis. The major lesion shows bone expansion at the metaphysis (arrow) with peritumoral edema and enhancement (curved arrow). (**C**) Axial T1-weighted and (**D**) enhanced images show a peripherally enhancing major lesion (arrow) with cortical remodeling (curved arrow).

**Figure 10:**
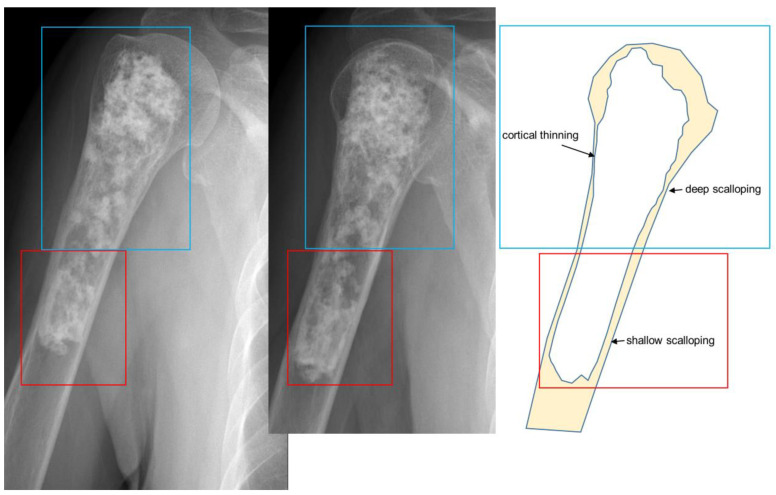
**Secondary central chondrosarcoma of the humerus in an 81-year-old woman.** The residual enchondroma in the red box (narrow scalloping) is combined with the additional features of chondrosarcoma in the blue box (cortical thinning and deep scalloping).

Secondary central chondrosarcomas present extended endosteal scalloping, cortical remodeling, cortical destruction, and periosteal reaction on plain radiographs, especially when compared to previous images of the underlying enchondroma [38,39]. On CT, the characteristic features of malignancy are lytic areas, endosteal scalloping on ≥2/3 of the cortex, or extension to soft tissue [38]. If one of the following criteria is present on MRI, malignant transformation of the underlying enchondroma can be assumed: cortical destruction, spontaneous pathologic fracture, periosteal reaction, peritumoral edema, and soft tissue mass [38] (Figure 9). However, the conversion of a solitary enchondroma to a chondrosarcoma remains controversial, mainly due to the need for radiologic evidence for an enchondroma showing its eventual transformation into chondrosarcoma over several decades of follow-up [18]. Recently, Brien et al. [18] reported the criteria for secondary central chondrosarcoma within a single lesion site at any time, even if no serial follow-up radiologic films are available. They reported that the features of conventional chondrosarcomas (endosteal scalloping, expansion of the affected bone, cortical thickening, and amorphous calcification) in association with the features of typical benign enchondromas (well-defined ring-and-arc calcifications) justify the diagnosis of secondary central chondrosarcoma even without prior demonstration of underlying silent enchondroma [18] (Figure 10). Most central chondrosarcomas are thought to be primary and constitute approximately 75% of all chondrosarcomas. However, remnants of pre-existing enchondromas were found in 40% of central chondrosarcomas, suggesting that most central chondrosarcomas could be secondary to a pre-existing enchondroma [18].

### 2.3. Third Stage: Central vs. Peripheral vs. Periosteal

Chondrosarcomas are also categorized as central, peripheral, or periosteal (juxtacortical), depending on the osseous location [1]. Central chondrosarcomas are intramedullary in origin (see Section 2.1), while peripheral chondrosarcomas arise within the cartilage caps of osteochondromas (see Section 2.2). Periosteal (juxtacortical) chondrosarcomas rarely (<2%) arise on the bone surface [5,17]. On gross pathologic examination, periosteal chondrosarcoma is covered by a fibrous pseudocapsule that is continuous with the periosteum [1]. Extrinsic erosion of the cortex is often present [1]. The histological appearance is identical to that of conventional central chondrosarcoma [1]. Periosteal chondrosarcomas most frequently affect adults in the 3rd to 4th decades of life and have a mild male predilection [1]. Of 59 cases reported in the literature, 29 (49%) were located in the femur, 14 (24%) in the humerus, and eight (14%) in the tibia, with more rarely reported sites including the ilium, fibula, and ribs [40,41,42]. Most cases involved a low-grade tumor with local recurrence rates of 13–28% and an overall disease-free 5-year survival of 83% [41,42].

**Figure 11:**
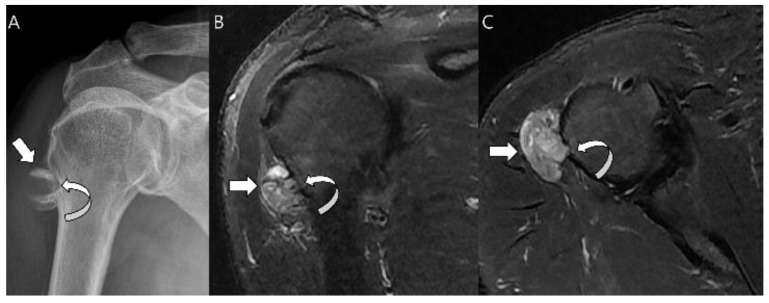
**Periosteal chondrosarcoma grade 1 of the humerus in a 66-year-old man.** (**A**) Radiograph shows a juxtacortical mass with Codman’s triangles (arrow) in the humerus. Note the associated cortical thinning (curved arrow). (**B**) Coronal and (**C**) axial T2-weighted images with fat suppression show a juxtacortical mass with high signal intensity and lobular margins (arrows). The mass has caused cortical erosion (curved arrow) but no evident marrow invasion.

Radiographs show a round to oval lobulated soft tissue mass on the surface of the bone, lifting the periosteum over the tumor as a fibrous pseudocapsule [1,5]. The underlying cortex is almost invariable, presenting as either thickened or thinned, while complete cortical destruction is rare [5]. A Codman triangle may be seen where the periosteum is lifted [1]. Typical chondroid matrix mineralization is usually present and metaplastic ossification is often seen to a variable extent [1]. The medullary canal is typically not involved, although extension has been observed on MRI [1,40,41] (Figure 11). Periosteal chondroma and periosteal osteosarcoma are the most difficult tumors to differentiate from periosteal chondrosarcoma [43,44]. Tumor size is the only differentiating feature between periosteal chondroma (median size 2.5 cm) and periosteal chondrosarcoma (median size 4 cm) [40]. Periosteal osteosarcomas and chondrosarcomas both contain cartilage, but chondrosarcomas show no osteoid formation on histological examination [41,43].

### 2.4. Fourth Stage: Conventional vs. Subtypes

Various histological subtypes of chondrosarcomas have been described, including conventional, mesenchymal, clear cell, and dedifferentiated [1]. Most chondrosarcomas are pathologically classified as conventional (80–85%; see Section 2.1). Several subtypes exist that differ in location, appearance, treatment, and prognosis [17]. These include clear cell (1–2%), mesenchymal (3–10%), and dedifferentiated (5–10%) chondrosarcomas [16].

**Figure 12:**
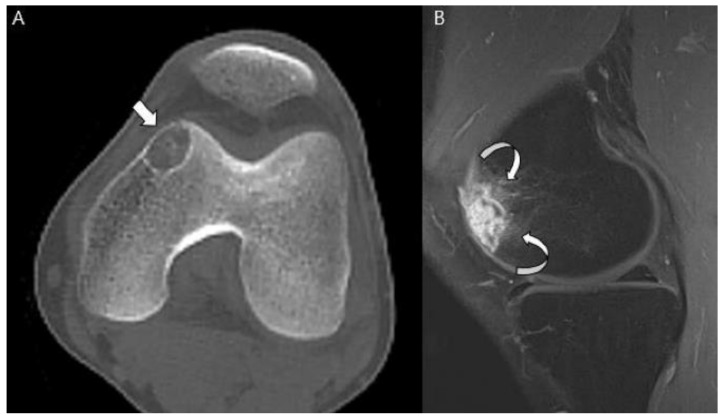
**Clear cell chondrosarcoma of the distal femur in a 31-year-old man.** (**A**) Axial CT scan shows an osteolytic lesion with a thin sclerotic margin at the distal femur (arrow). (**B**) Sagittal T1-weighted enhanced image with fat suppression shows a heterogeneously enhancing lesion with mild peritumoral enhancement at the distal femoral epiphysis (curved arrows).

Clear cell chondrosarcomas are low-grade variants characterized by an epiphyseal location in long bones [45]. On histological analysis, these lesions have numerous cells with abundant clear vacuolated cytoplasm [1,5]. Patients are most commonly affected in the 3rd to 5th decades of life [1]. Long bones are affected in 85–90% of cases with the proximal femur (68%) and proximal humerus (23%) the most commonly involved long bones [45]. Radiographs reveal a predominantly lytic epiphyseal lesion with distinct sclerotic margins that simulate a benign lesion [5,45] (Figure 12). Matrix mineralization is not as frequently apparent in clear cell chondrosarcomas (approximately 30% of cases) as in conventional chondrosarcomas [46,47,48]. In approximately 30% of cases, mild bone expansion may be apparent, but soft tissue extension is rare (<10% of cases) [1,5]. Because of their epiphyseal location, clear cell chondrosarcomas can be difficult to distinguish from chondroblastomas [1]. Clinically, clear cell chondrosarcomas usually present one or two decades later than chondroblastomas [18]. On MRI, clear cell chondrosarcomas are heterogeneous due to areas of hemorrhage or cystic changes [45]. Peritumoral edema is unusual and always mild as opposed to that in chondroblastoma [45].

Mesenchymal chondrosarcomas are a rare high-grade variant that has a strong tendency to metastasize. They can originate from either bone or soft tissue [1]. The characteristic histological feature of this tumor type is a bimorphic pattern characterized by differentiated cartilage admixed with solid highly cellular areas composed of undifferentiated small round cells [1]. In the undifferentiated areas, small, round cells typically simulate Ewing’s sarcoma and have a hemangiopericytomatous vascular pattern [49,50]. The prognosis of mesenchymal chondrosarcomas is poor, and they present in a younger age group than conventional chondrosarcomas (mean age ~25 years) [5]. In contrast to conventional chondrosarcomas, mesenchymal chondrosarcomas most commonly involve the axial skeleton; for example, the craniofacial region [1]. Radiographs usually show aggressive bone destruction with a moth-eaten to permeative bone pattern and an ill-defined periosteal reaction [51,52]. The tumor is often very large with extensive extraosseous components [1]. CT typically shows chondroid mineralization, and the lesion may appear heavily calcified, but more commonly shows “finely stippled” calcification [53]. Mesenchymal chondrosarcomas have a different pattern of contrast enhancement than conventional chondrosarcomas on MRI; often, diffuse and typical chondroid septal and peripheral enhancement is lacking [1]. Some areas show low-signal, serpentine, high-flow vessels, a feature not seen in other chondrosarcomas [1]. The diagnosis of mesenchymal chondrosarcoma is suggested by an aggressive osseous lesion with subtle chondroid matrix mineralization and an intermediate signal on T2WI (lower than that of conventional chondrosarcoma), with more dramatic enhancement than expected with conventional chondrosarcoma [1].

**Figure 13:**
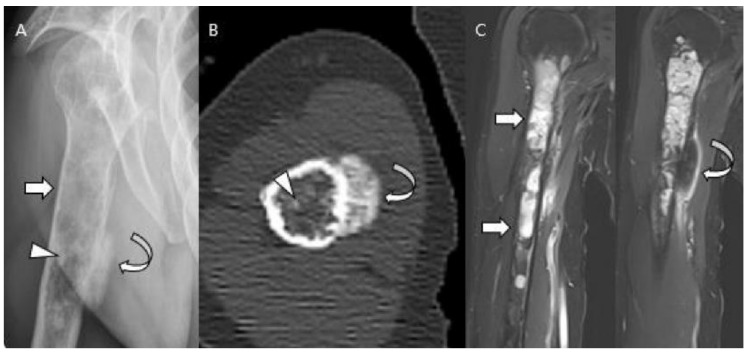
**Dedifferentiated chondrosarcoma of the humerus in a 54-year-old man.** (**A**) Plain radiograph shows an extensive mixed lytic and sclerotic lesion in the humerus with endosteal scalloping (arrow). Note the chondral-type mineralization in the intramedullary cavity (arrowhead) and the densely osteoid-type mineralization at the juxtacortical area (curved arrow). (**B**) Axial CT scan also reveals the intramedullary chondral-type (arrowhead) and the juxtacortical dense osteoid-type (curved arrow) mineralization. (**C**) Coronal T2-weighted images with fat suppression show high signal intramedullary lesion (arrows) with osteoblastic extraosseous extension (curved arrow), suggesting a dedifferentiated component of osteosarcoma.

Dedifferentiated chondrosarcoma is characterized by a conventional low-grade chondrosarcoma with an abrupt transition to foci that have dedifferentiated into a higher-grade, more aggressive component [1]. The non-cartilaginous portion is most frequently conventional osteosarcoma (70%) and less commonly malignant fibrous histiocytoma or fibrosarcoma [1,5]. Dedifferentiation can occur in 10–20% of conventional chondrosarcomas [1]. Patients with dedifferentiated chondrosarcomas are older than those with conventional lesions, usually 50–70 years of age (mean age: approximately 60 years) [54,55,56]. Dedifferentiated chondrosarcomas have a poor prognosis. A multicenter review of 337 patients reported that 21% had metastases at the time of diagnosis and the survival of these patients was 10% at 2 years [18,57]. The sites of involvement parallel those of conventional intramedullary chondrosarcoma, with common locations including the femur (35% of cases), pelvis (29%), humerus (16%), scapula (6%), rib (6%), and tibia (5%) [54,55,56]. The radiographic features of dedifferentiated chondrosarcomas are tumor bimorphism including aggressive bone destruction with extraosseous soft tissue extension, associated with an underlying cartilaginous lesion [17]. The imaging findings vary depending on the areas of high-grade transformation [1,58]. Tumors can be classified into three types based on radiographic findings: type 1, radiographic features the same as those of a central chondrosarcoma, with the addition of a suspected region with dedifferentiation; type 2, the tumor resembles the underlying benign enchondroma, but with destructive changes and/or a large soft tissue mass; and type 3, high-grade destructive lesions of the bone without signs of a cartilaginous component [56]. CT and MRI may reveal two distinct areas with differing intrinsic characteristics [1] (Figure 13). This bimorphic pattern is valuable in targeting the high-grade region during image-guided needle biopsy [59].

**Figure 14:**
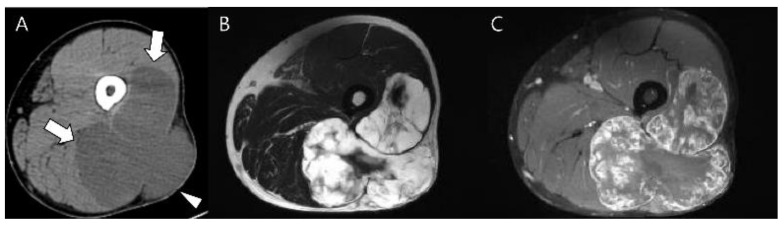
**Extraskeletal myxoid chondrosarcoma of the right thigh in a 46-year-old man.** (**A**) Axial CT scan reveals a lobulated, low-density soft tissue mass (arrows) without chondral-type mineralization between the vastus lateralis and biceps femoris muscles extending to the subcutaneous fat layer (arrowhead). (**B**,**C**) Axial T2-weighted and T1-weighted enhanced images show a soft tissue mass with high signal intensity and peripheral rim and septal enhancement.

Myxoid chondrosarcomas are now generally accepted as prominent myxoid changes of high-grade conventional chondrosarcomas [17]. However, extraskeletal myxoid chondrosarcoma (EMC) is a disease entity distinct from chondrosarcoma of the bone; these soft tissue sarcomas most commonly arise in the lower extremities [60,61] (Figure 14). The term “chondrosarcoma” used to describe EMC is a misnomer because well-formed hyaline cartilage is found only in a minority of EMCs, and S100 expression (which is present in all or most chondrosarcomas) is often very focal or absent [62,63]. The 2020 WHO classification categorizes EMC as “tumors of uncertain differentiation” [64]. Myxoid chondrosarcomas of the bone are also not designated as unique entities; rather, these tumors should be regarded as myxoid variants of conventional chondrosarcomas [7].

## 3. Diagnostic Dilemma of Chondrosarcoma Classification

### 3.1. Distinction between Enchondroma and ACT

The differentiation between enchondromas and ACTs is crucial, as ACTs require curettage and watchful imaging follow-up, whereas most enchondromas require neither treatment nor follow-up [65]. Many imaging findings allow the differentiation between enchondromas and ACT, including cortical destruction, extraosseous soft tissue mass extension, periosteal reaction, size ≥ 5 cm, and endosteal scalloping (>2/3 of the cortical thickness) [66,67,68]. However, differentiating ACTs from enchondromas is challenging due to the lack of a gold standard for the diagnosis of ACT on histopathology [69,70]. While the presence of permeation and entrapment of pre-existing trabecular bone on histopathology are diagnostic for ACT, they may also result in a diagnostic conundrum, especially in cartilaginous lesions showing borderline imaging features in young patients, such as endosteal scalloping of approximately 50% of the cortex, lesion length of approximately 5 cm, or a change in the mineralization pattern with a lack of permeation [8]. In the absence of specific diagnostic criteria for histopathology, the differentiation between these two disease entities is often established by a consensus between radiologic, pathologic, and clinical findings [24]. 

**Figure 15:**
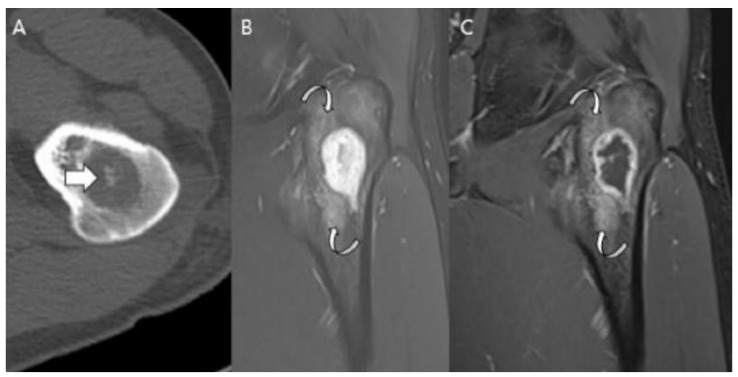
**Atypical cartilaginous tumor of the proximal femur in a 22-year-old man.** (**A**) Axial CT scan reveals a low-density intramedullary mass with chondral-type mineralization (arrow) in the proximal femur. (**B**,**C**) Coronal T2-weighted fat-suppressed and T1-weighted enhanced images show an intramedullary mass of 3 cm in size with high signal intensity and the peripheral rim and septal enhancement. Note the peritumoral edema with enhancement (curved arrows).

The differentiation between enchondromas and ACT has been researched extensively because there remains low reliability in the clinical, radiological, and pathological distinctions between these two disease entities [70]. Choi et al. [66] identified some MRI features helpful for differentiating ACT from enchondroma, including the presence of a predominantly intermediate signal matrix on T1WI, multilobulated enhancement pattern on enhanced T1WI, cortical destruction, soft tissue mass, epiphyseal or flat bone involvement, and peritumoral edema (Figure 15), which favored a diagnosis of ACT. De Coninck el al. [71] evaluated the role of dynamic contrast-enhanced MRI (DCE-MRI) for the differentiation of enchondromas from chondrosarcomas and found that enhancement within the tumor, which was two times greater than that to muscle, combined with a 76° slope of the uptake curve, showed 100% sensitivity and 63% specificity for the detection of chondrosarcomas. However, the role of DCE-MRI in the differentiation of enchondroma from ACT remains ambiguous due to the lack of clear diagnostic histopathological criteria and the inclusion of low-grade and high-grade chondrosarcomas in previous studies [8]. In addition, diffusion-weighted imaging (DWI) is of no value in differentiating between enchondroma and ACT [72]. Studies quantifying tumor heterogeneity, including those applying MRI texture analysis, have shown improved diagnostic accuracy for the differentiation of benign and malignant cartilaginous tumors [68,73]. Assessing heterogeneity with imaging could provide important information on tumor characterization and might be a non-invasive biomarker for discrimination between tumor grades [68]. Pan et al. [74] developed three clinical radiomics nomograms to predict the malignancy risk of cartilaginous tumors based on radiomic signatures and clinical risk factors. All three nomograms demonstrated high performance for the differentiation of chondrosarcoma from enchondroma based on T1WI, fat-suppressed T2WI, and T1WI + T2WI fat-suppressed sequences with better accuracy than those of morphologic MRI analysis by musculoskeletal radiologists. 

### 3.2. Biopsy or Follow-Up? Questions for Incidental Cartilage Lesions in the Long Bones

The increased use of MRI, which is now available in most healthcare systems, has resulted in the increased incidental identification of cartilage lesions in the long bones. Most of these lesions do not undergo biopsy and there is, typically, no histological confirmation of the diagnosis [75]. This may result in overtreatment of an enchondroma radiographically diagnosed as ACT or undertreatment if ACT is radiographically diagnosed as an enchondroma and the patient is erroneously discharged without follow-up [76]. However, a universal consensus on the management of these lesions is lacking; some centers recommend curettage, while others suggest surveillance with imaging [77,78]. Many authors have proposed radiographic follow-up protocols instead of biopsy for lesions without signs of local aggressiveness (cortical destruction and soft tissue extension), resulting in lower morbidity and costs [75,76,79,80]. The most recent studies on cartilaginous tumors have shifted toward active surveillance of ACTs to avoid unnecessary surgeries [80,81,82].

**Figure 16:**
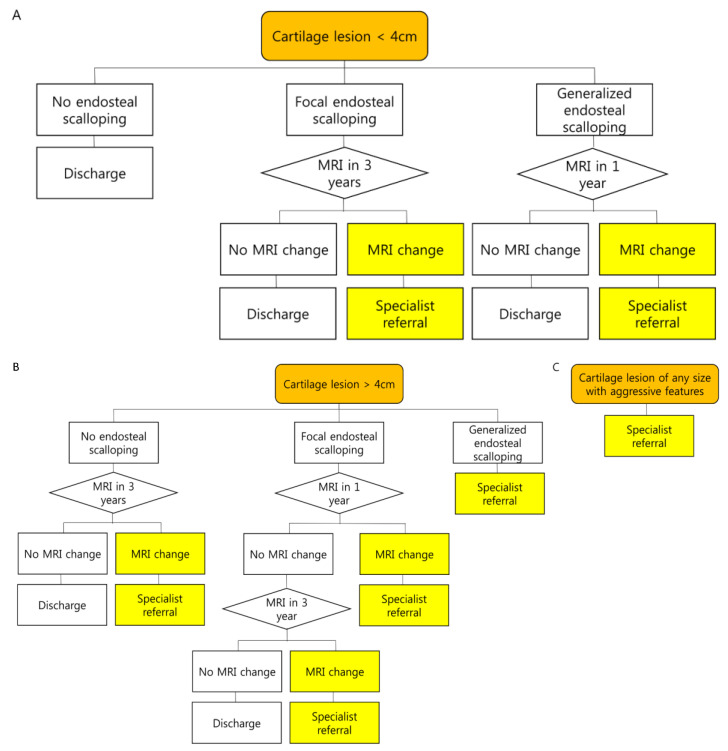
Birmingham Atypical Cartilaginous Tumor Imaging Protocol applied to cartilage lesions in the proximal humerus and around the knee [79]. (**A**) Cartilage lesion < 4 cm, focal endosteal scalloping ≤10% or 36° of lesion circumference on the axial image with the greatest involvement; generalized endosteal scalloping ≥10% or 36° of lesion circumference on the axial image with the greatest involvement; MRI change = increase in longitudinal length of lesion ≥1 cm and/or development of aggressive features including increasing endosteal scalloping. (**B**) Cartilage lesion > 4 cm. (**C**) Cartilage lesion of any size with aggressive features (bone expansion and/or cortical thickening, periostitis, cortical destruction, and soft tissue mass).

One study suggested distinguishing “active” lesions from “quiescent” lesions and recommended biopsy for the former (endosteal scalloping >2/3 of the cortex and >2/3 the length of the tumor, cortical thickening, and bone expansion) and radiological follow-up for the latter (in the absence of active findings) [77]. Kumar et al. [75] divided patients into “active” and “latent” groups based on the total growth of the cartilage lesion and advocated for biopsy in the active group with total growth > 6 mm, with surveillance with MRI every 3 years in the latent group. However, consensus evidence is lacking in the literature regarding follow-up frequency or duration, and no recommendations have been suggested for optimal imaging protocols. Deckers et al. [76] recommended annual MRI at least 2 years after diagnosis; if the findings remain stable, the frequency of MRI could be reduced to every 2 or 3 years. Herget et al. [38] recommended annual clinical and annual or biannual MRI for asymptomatic lesions > 5–6 cm and annual clinical and biannual imaging studies (radiographs or MRI if any doubts) for asymptomatic lesions < 5–6 cm. Patients with cartilage lesions ≤ 4 cm long with no endosteal scalloping can be discharged, with instructions to contact the hospital in case of new or increased pain [79]. In contrast, surgery is advised for tumors showing any aggressive features during follow-up, with curettage the preferred treatment for ACT [83]. Needle biopsies should not be recommended because they do not clearly differentiate enchondromas from ACT [83]. Several management protocols have been proposed [65,75,80,84]. We introduced the Birmingham Atypical Cartilaginous Tumor Imaging Protocol (Figure 16), which can be applied to cartilage lesions in the proximal humerus and around the knee [79]. As this protocol is only a guideline and has not been clinically validated, we cannot accept responsibility for any issues that may arise from its use [79].

### 3.3. Distinction between ACT/CS1 and High-Grade Chondrosarcoma

**Figure 17:**
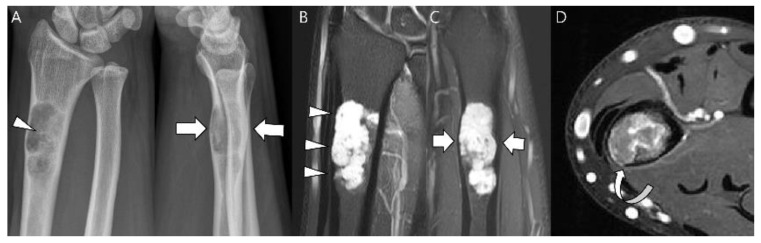
**A 35-year-old man presenting with wrist pain.** (**A**) Plain radiographs reveal a lobulated lytic lesion with chondroid matrix mineralization (arrowhead) and bone expansion (arrows) in the distal radius. (**B**,**C**) Coronal and sagittal T2-weighted fat-suppressed images show an intramedullary high signal mass with deep and extensive endosteal scalloping (arrowheads) and bone expansion (arrows). (**D**) Axial T1-weighted enhanced image shows peripheral rim and septal enhancement. Note the volar cortical thinning or defect (curved arrow). This lesion was noted as an atypical cartilaginous tumor at the initial incisional biopsy but was revealed as chondrosarcoma grade 2 at extended curettage.

With the increasing incidence of ACT, the need for clear radiologic criteria to differentiate ACT from high-grade chondrosarcoma has become more important due to the different treatment options and prognoses [85]. High-grade chondrosarcoma requires wide resection with free surgical margins, whereas ACTs located in the long bones can be treated with intralesional curettage or regular follow-up [76]. However, the grading of chondrosarcoma based on imaging findings has shown low reliability; many diagnostic biopsies are unreliable owing to the heterogeneous composition of chondroid tumors (Figure 17) [69,86].

**Figure 18:**
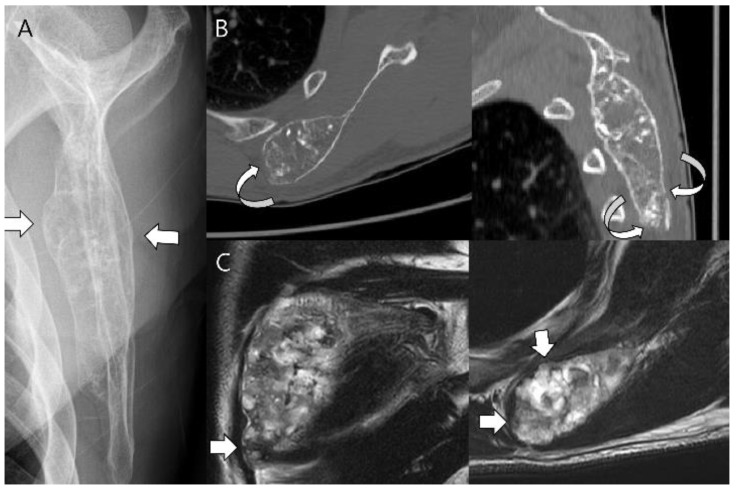
**Chondrosarcoma grade 2 of the scapula in a 58-year-old man.** (**A**) Plain radiograph shows a lobulated intramedullary mass with chondral-type mineralization and bone expansion (arrows) in the scapular body. (**B**) Axial and sagittal CT scans show a large intramedullary mass with cortical destruction (curved arrows). (**C**) Coronal and axial T2-weighted images show focal extraosseous soft tissue masses (arrows).

**Figure 19:**
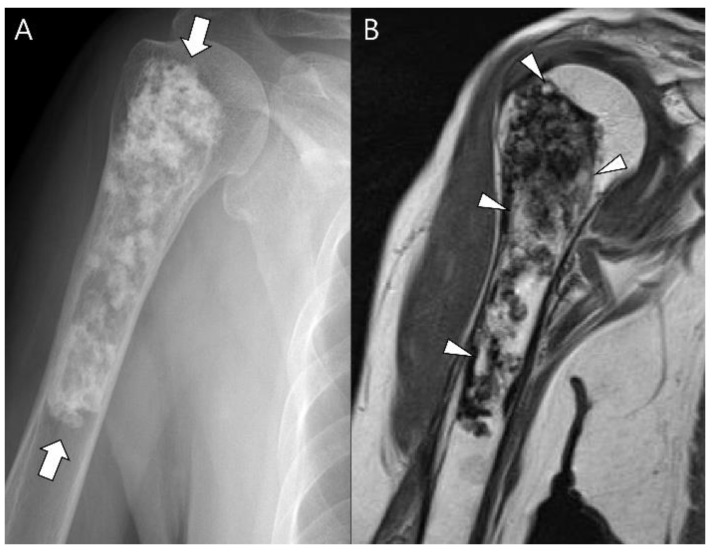
**Atypical cartilaginous tumor of the humerus in an 81-year-old woman.** (**A**) Plain radiograph demonstrates an intramedullary mass with prominent chondroid matrix mineralization (arrows) in the humerus. (**B**) Coronal T1-weighted image shows a lobulated intramedullary mass with areas of entrapped medullary fat (arrowheads).

High-grade chondrosarcoma may more often present with the following radiographic characteristics: moth-eaten or permeative bone destruction, less extensive matrix mineralization, loss of entrapped fatty marrow, cortical destruction, and a more aggressive periosteal reaction compared to ACT [1,85]. In addition, the histologic grades of lesions arising in the bones are poorer than those in the appendicular skeleton [87]. MRI is the modality of choice for identifying not only these radiographic features, but also the features of high-grade lesions, such as abundant (>50%) myxoid matrix, cortical destruction, soft-tissue extension, peritumoral edema, and periostitis (Figure 18) [88,89]. Jain et al. [87] reported that bone expansion did not differentiate between ACT/CS1 and high-grade chondrosarcoma unless the cortex was intact. Hemorrhagic necrosis and intra-articular extension are features of high-grade chondrosarcoma [87]. A biphasic pattern with a high-grade non-chondral sarcoma located adjacent to a typical chondral tumor is a characteristic feature of dedifferentiated chondrosarcoma [90] (Figure 13). Conversely, entrapped fat within the tumor and a characteristic lobular tumor morphology are highly indicative of ACT (Figure 19) [85,91].

Beyond CT and MRI, DCE-MRI can aid in the diagnosis of high-grade chondrosarcoma because it can reveal areas of fast enhancement due to richly vascularized intralesional septations [71,92]. However, DWI cannot differentiate low-grade lesions from high-grade chondrosarcomas [72]. Thus, novel tools for the objective grading of chondrosarcomas have recently been introduced, including texture analysis [73,93] and radiomics [94] with quantitative analysis. Deng et al. [93] reported that CT-based texture analysis showed potential for the grading of cartilaginous tumors in long bones. Gitto et al. [94] reported that their machine-learning approach showed satisfactory diagnostic performance for the classification of low-to-high-grade cartilaginous bone tumors based on radiomic features extracted from unenhanced MRI. One systemic review concluded that radiomics may allow the optimization of surgical decision making in chondrosarcoma despite weak evidence or insufficient study quality [95].

## 4. Current Treatments and Management

The therapeutic approach for chondrosarcomas is determined by the location and histologic grade. Surgical excision is the primary treatment for chondrosarcomas. Low-grade central chondrosarcoma can be treated with intralesional curettage, burring, and surgical adjuvant application such as hydrogen peroxide [96]. Tumors with extraosseous soft tissue extension, larger tumors, and axial skeleton tumors require wide excision. Wide en-bloc excision is the surgical approach of choice for intermediate or high-grade chondrosarcomas [97]. However, many patients show inoperable conditions at diagnosis or recur with metastatic disease, with more than 10% of recurrence cases showing a higher grade of malignancy than the first diagnosed grade [98].

Chemotherapy is usually ineffective in conventional and clear cell chondrosarcomas [97]. However, it may play a role in dedifferentiated chondrosarcomas containing high-grade spindle cell components [99]. A systematic review of 31 published studies suggested that adjuvant chemotherapy combined with surgical resection significantly improves disease-free survival in dedifferentiated chondrosarcomas compared to surgery alone [100]. In a non-randomized clinical cohort, adjuvant anthracyclin-based combination chemotherapy showed modest efficacy against mesenchymal chondrosarcomas [101].

Chondrogenic tumors are generally considered radioresistant because radiation-induced cytotoxicity requires actively dividing cells. Chondrogenic tumors are characterized by slow growth and a relatively low proportion of dividing cells [97]. However, radiation therapy can be administered after incomplete resection of high-grade conventional, dedifferentiated, or mesenchymal chondrosarcomas, with potential curative intent to maximize local control. Definitive radiation may also be indicated for palliative purposes [102].

## 5. Targets and Novel Treatment Options

Chondrosarcomas are poorly responsive to chemotherapy and radiation therapy, resulting in high morbidity and mortality [103]. Therefore, there is an urgent need to expand treatment options. Developing an efficient treatment strategy requires a better understanding of the molecular survival pathways involved in chondrosarcomas and their chemotherapy and radiation resistance mechanisms [104]. Chondrosarcoma subtypes differ at the molecular genetic level (Table 2) [105]. Recent studies have suggested several promising biomarkers and therapeutic targets for chondrosarcomas, with better understanding of chondrosarcoma genomic alterations and biology [103,105,106,107,108,109,110]. As shown in Table 2, the signaling pathways underpinning chondrosarcoma genesis such as IDH1/2 mutations, CDKN2A/B deletions, and TP53 mutations can be potential therapeutic targets [105]. The angiogenesis pathway is a potential effective target for preventing the growth and spread of chondrosarcoma [105]. Conventional chondrosarcomas are characterized by activation and/or overexpression of platelet-derived growth factor receptors PDGFR-alpha (PDGFRA) and PDGFR-beta (PDGFRB), and efforts to develop antiangiogenic therapies have produced many agents such as small molecule tyrosine kinase inhibitors and fully human monoclonal antibodies which affect angiogenesis [111]. Also, a multitargeted approach against multiple antiapoptotic proteins such as Bcl-2 (B-cell leukemia/lymphoma 2), Bcl-xL (Bcl-2 like 1), and XIAP (x-linked inhibitors or apoptosis) upregulated in chondrosarcomas can have a strong therapeutic potential to enhance the efficacy of radiation and chemotherapy [104]. These findings prompted research on the therapeutic efficacy of molecular-targeting therapies [103,112].

## 6. Conclusions

Chondrosarcomas are a heterogeneous group of malignant bone tumors that produce a chondroid (cartilaginous) matrix. Their clinical behaviors vary according to the histologic grade. The WHO defines these lesions as benign, intermediate, or malignant cartilaginous tumors. While most tumors are indolent, with a low potential for metastasis, some are aggressive, with a poor prognosis. Clinical management is guided by imaging findings, histopathological grading, and chondrosarcoma subtypes. Choosing the most appropriate diagnostic technique for grading chondroid tumors remains difficult because each modality has its own value; beyond CT and MRI, DCE-MRI supports chondrosarcoma grading, and new tools for quantitative analysis—including texture analysis and radiomics—have shown satisfactory diagnostic performance for chondrosarcoma classification. A limited range of treatment options exists for chondrosarcomas, including surgery and chemotherapy, and more therapeutic targets are needed. Multidisciplinary discussions of all modalities should be combined to determine the best treatment approach. 

## Figures and Tables

**Table 1:** 2020 WHO classification of chondrosarcomas [7,13].

Entity		Remarks
Conventional chondrosarcomas	Central atypical cartilaginous tumor (ACT)/chondrosarcoma grade 1 (CS1)	De novo or secondary (possible precursor: enchondroma)
	Secondary peripheral ACT/CS1	Precursor: osteochondroma
	Central chondrosarcoma grades 2 and 3 (CS2,3)	De novo or secondary (possible precursor: enchondroma)
	Secondary peripheral CS2,3	Precursor: osteochondroma
	Periosteal chondrosarcoma	
Rare subtypes	Dedifferentiated chondrosarcoma	Precursor: conventional chondrosarcoma
	Mesenchymal chondrosarcoma	
	Clear cell chondrosarcoma	

**Table 2:** Chondrosarcoma types and respective molecular features.

Chondrosarcoma (CS) Type	Molecular Features
Conventional central CS	IDH1/2 mutations COL2A1 mutations CDKN2A/B deletions
Conventional peripheral CS	EXT1/2 mutations
Conventional periosteal CS	Hedgehog pathway
Dedifferentiated CS	IDH1/2 mutations TP53 mutations PD-L1 expression
Mesenchymal CS	HEY1–NCOA2 fusion
Clear cell CS	No evidence of mutations

## Data Availability

Not applicable.
